# Distribution of Serum Total Protein in Elderly Chinese

**DOI:** 10.1371/journal.pone.0101242

**Published:** 2014-06-26

**Authors:** Chang-Rong Tian, Li Qian, Xiao-Zhu Shen, Jia-Jing Li, Jiang-Tao Wen

**Affiliations:** 1 Department of Geriatrics, The Second People’s Hospital of Lianyungang, Xinpu, China; 2 Department of Microbiology Laboratory, The Second People’s Hospital of Lianyungang, Xinpu, China; 3 Department of Electronic and Information Engineering Center, The Second People’s Hospital of Lianyungang, Xinpu, China; 4 Department of Clinical Science Experiment Center, The Second People’s Hospital of Lianyungang, Xinpu, China; University of Louisville, United States of America

## Abstract

The serum total protein levels of the elderly possibly decrease gradually with aging. However, serum total protein levels are not suitable as a uniform reference standard for the elderly at different ages and genders. Thus, we investigated the total serum protein distribution in different gender and age groups of 11,453 elderly individuals aged ≥60 years and without liver or renal disease from Lianyungang, Jiangsu, China. The total protein levels (TPL) of these individuals exhibited normal distribution (Z = 1.206, *P = *0.109), whereas the reference range (95% CI) was 54.1 g/L to 82.3 g/L. TPL was higher in females than in males for those aged between 60 and 75 years, whereas no significant difference was observed for those aged between 80 and 95 years. TPL was negatively correlated with age in males (r = −0.1342, *P*<0.05), females (r = −0.304, *P*<0.05), and the total group (r = −0.2136, *P*<0.05). TPL also decreased with aging and showed a faster rate in women than in men. These results indicated that an appropriate range of serum total protein based on age and gender differences should be used for clinical applications.

## Introduction

According to the sixth national census released by the China National Bureau of Statistics until November 1, 2010, 0∶00 hours, the number of Chinese aged ≥60 years was 178 million and accounts for 13.26% of the total population. China has entered the phase of an aging society, on which the elderly, as a special group with its unique pathological and physiological characteristics, has been greatly considered by medical researchers [1]. In the elderly, serum total protein levels possibly decrease gradually with age because the volume and number of liver cells decrease with age; as a result, serum total protein components are decreased. The adult standard range of 64 g/L to 83 g/L serum total protein is used as reference for the elderly [2], [3]. However, this uniform reference standard is not suitable for the elderly at different ages and genders; an accurate reference range of serum total protein level is required to assess the clinical conditions of elderly patients, guide diagnosis and treatment measures, and provide reference for prognosis [4]. Thus, we investigated the total serum protein distribution of 11,453 elderly individuals aged ≥60 years from Lianyungang, Jiangsu, China.

Serum total protein is a complex mixture of various proteins that can be separated using different methods. Plasma albumin, alpha 1-globulin, alpha 2-globulin, beta globulin, fibrinogen, prothrombin, and other clotting factors are synthesized by the liver. Gamma globulin is mainly produced by plasma cells [5].

Serum protein content can be determined using the Biuret test, in which peptide bonds, which are found in the same frequency as amino acids in peptides, interact with bivalent copper ions (Cu^2+^) to form blue/purple complexes in alkaline solutions. Protein content can be accurately measured by comparing a test sample with a standard solution of known concentration [6], [7]. This method also exhibits several advantages, including convenience and good repeatability; in this method, a similar reaction occurs between albumin and globulin and less interfering substances that cause low sensitivity are present [8].

## Materials and Methods

### 1. Ethics Statement

This study was approved by the ethics committee of The Second People’s Hospital of Lianyungang, China. The participants provided their verbal informed consent to participate in this study and this consent was recorded in an electronic spreadsheet as approved by the ethics committee of The Second People’s Hospital of Lianyungang, China.

### 2. Collection of Samples

A total of 14,453 elderly individuals (6,103 females and 8,350 males) from Lianyungang, Jiangsu, China were recruited to participate in the study, which was performed from January 2009 to March 2012. The age of the subjects ranged from 60 years to 101 years with an average age of 72.176±7.67 years. The proportion of the subjects aged ≥80 years was 19.6%. The inclusion criteria were described as follows: all of the subjects were essentially healthy without evidence of any chronic illness, including hepatic, renal, thyroid, and severe infectious diseases, malnutrition, tuberculosis, hyperthyroidism, or malignancy. All of the subjects were grouped according to gender and age, with group intervals of 5 years.

### 3. Biochemical Analysis

The subjects were instructed to fast overnight; afterward, a venous blood sample (3 mL) was drawn from each subject who was in a sitting position between 08∶00 and 09∶00 h. Serum total protein levels were measured immediately by using an automatic biochemistry analyzer (Dimension RXLMAX_HM, Siemens AG, Munich, Germany) in the Clinical Science Experiment Center, the Second People’s Hospital of Lianyungang, China. The measurement was performed by observing a Biuret reaction using a total protein reagent kit (Siemens AG, Munich, Germany) [9]–[11].

### 4. Statistical Methodology

The levels of the quantitative variables were presented as mean ± SD. The normal distribution of the total protein levels of 14,453 elderly individuals was determined using Kolmogorov-Smirnov *Z* test. The distribution of the total protein levels of each age group of males, females, and total subjects was statistically evaluated on the basis of frequencies, and 95% confidence interval (CI) was considered as the reference value. To compare the differences between each gender of diverse ages, we performed an independent samples *t*-test. The association of total protein levels with age in males, females, and the total subjects was analyzed by linear regression, and the associated graph was obtained. All of the tests were two-sided and *P*<0.05 was considered significant. Data were analyzed using SPSS 16.0 (SPSS 16.0, Chicago, Illinois, USA).

## Results

### 1. Normal distribution of serum total protein

The serum total protein in all of the elderly individuals aged ≥60 years showed a normal distribution as indicated in the result of Kolmogorov-Smirnov *Z* test (*Z* = 1.206 and *P* = 0.109).

### 2. Serum total protein reference range and distribution

**Figure 1:**
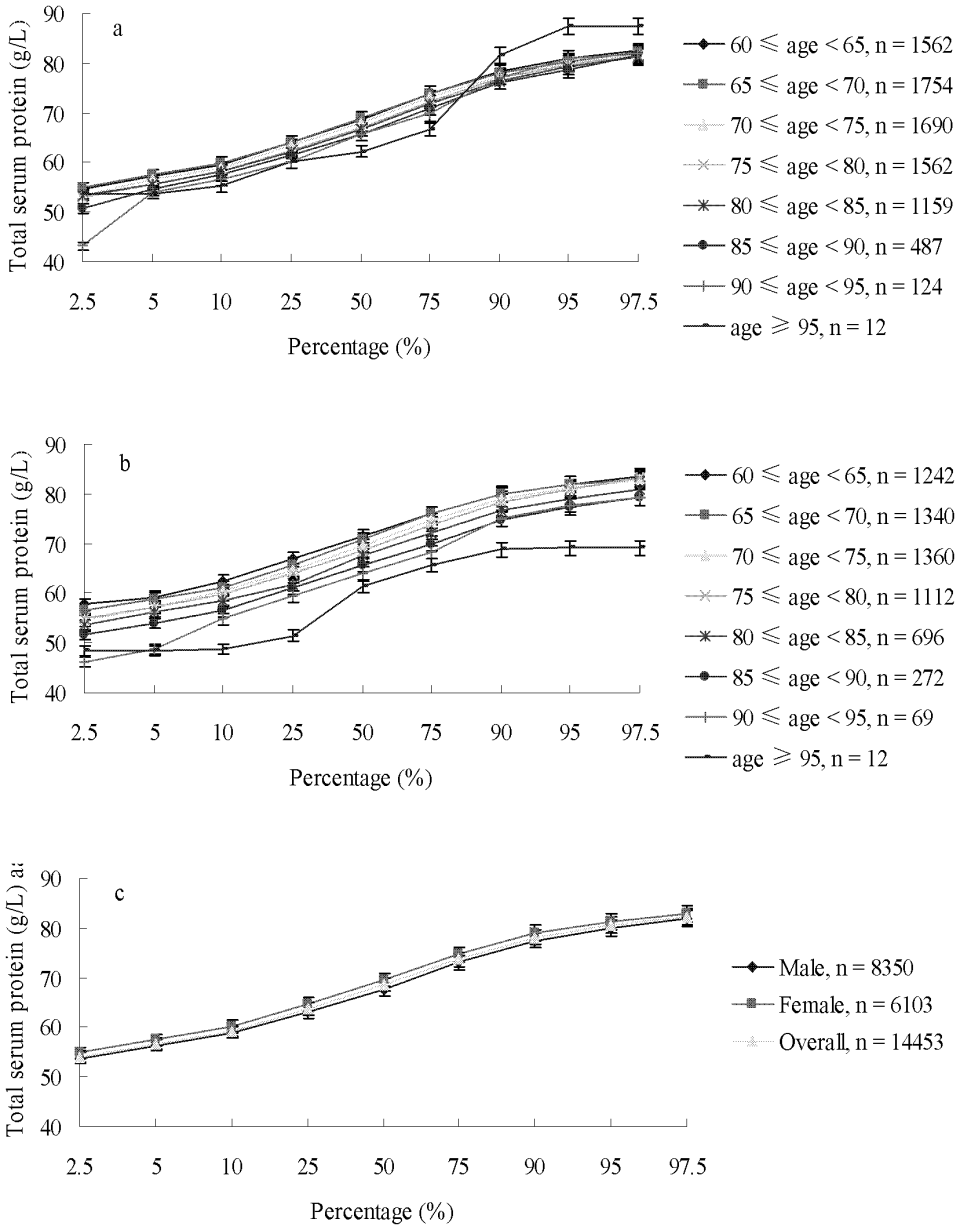
The percentage distribution of serum total protein in male (a), female (b), and total (c) groups aged ≥60.

The reference range (95% CI) of the serum total protein in all of the elderly subjects was 54.1 g/L to 82.3 g/L (Figure 1). The serum total protein levels of male subjects were 54.6 g/L to 82.6 g/L, 54.9 g/L to 82.3 g/L, 53.3 g/L to 82.0 g/L, 53.0 g/L to 81.7 g/L, 53.3 g/L to 81.1 g/L, 50.8 g/L to 81.5 g/L, 43.1 g/L to 82.0 g/L, and 53.6 g/L to 87.4 g/L in the respective age groups of 60, 65, 70, 75, 80, 85, 90, and 95 years (Figure 1a). By comparison, the serum total protein levels of the elderly females were 57.7 g/L to 83.5 g/L, 56.6 g/L to 82.9 g/L, 54.5 g/L to 82.7 g/L, 54.8 g/L to 83.1 g/L, 53.5 g/L to 81.0 g/L, 51.7 g/L to 79.4 g/L, 46.1 g/L to 79.2 g/L, and 48.4 g/L to 69.1 g/Lin the respective age groups of 60, 65, 70, 75, 80, 85, 90, and 95 years (Figure 1b).

### 3. Comparative analysis of the serum total protein between different age groups of elderly males and females

**Figure 2:**
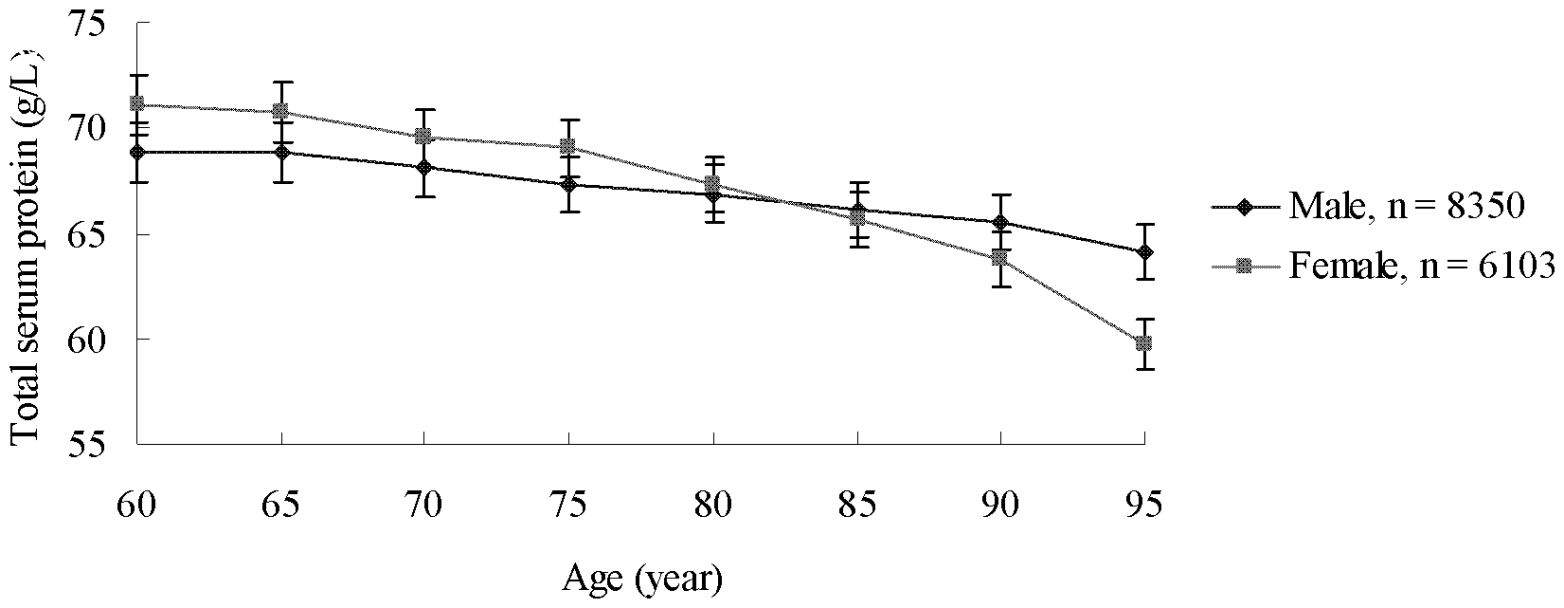
Effect of age on total protein levels in different gender groups aged ≥60.

The serum total protein levels were relatively higher in females than in males in the age groups of 60, 65, 70, and 75 years; by contrast, no significant difference was observed in the age groups of 80, 85, 90, and 95 years (Figure 2).

### 4. Decreasing trends of serum total protein levels with age in different gender groups

The decreasing level of serum total protein in terms of age occurred at a faster rate in the female group than in the male group. The serum total protein levels were relatively higher in elderly females than in elderly males before they reached the age of 79 years; by comparison, no significant difference was observed in the decreased serum total protein levels of elderly individuals aged ≥80 years (Figure 2).

### 5. Linear regression of serum total protein levels with age

**Figure 3:**
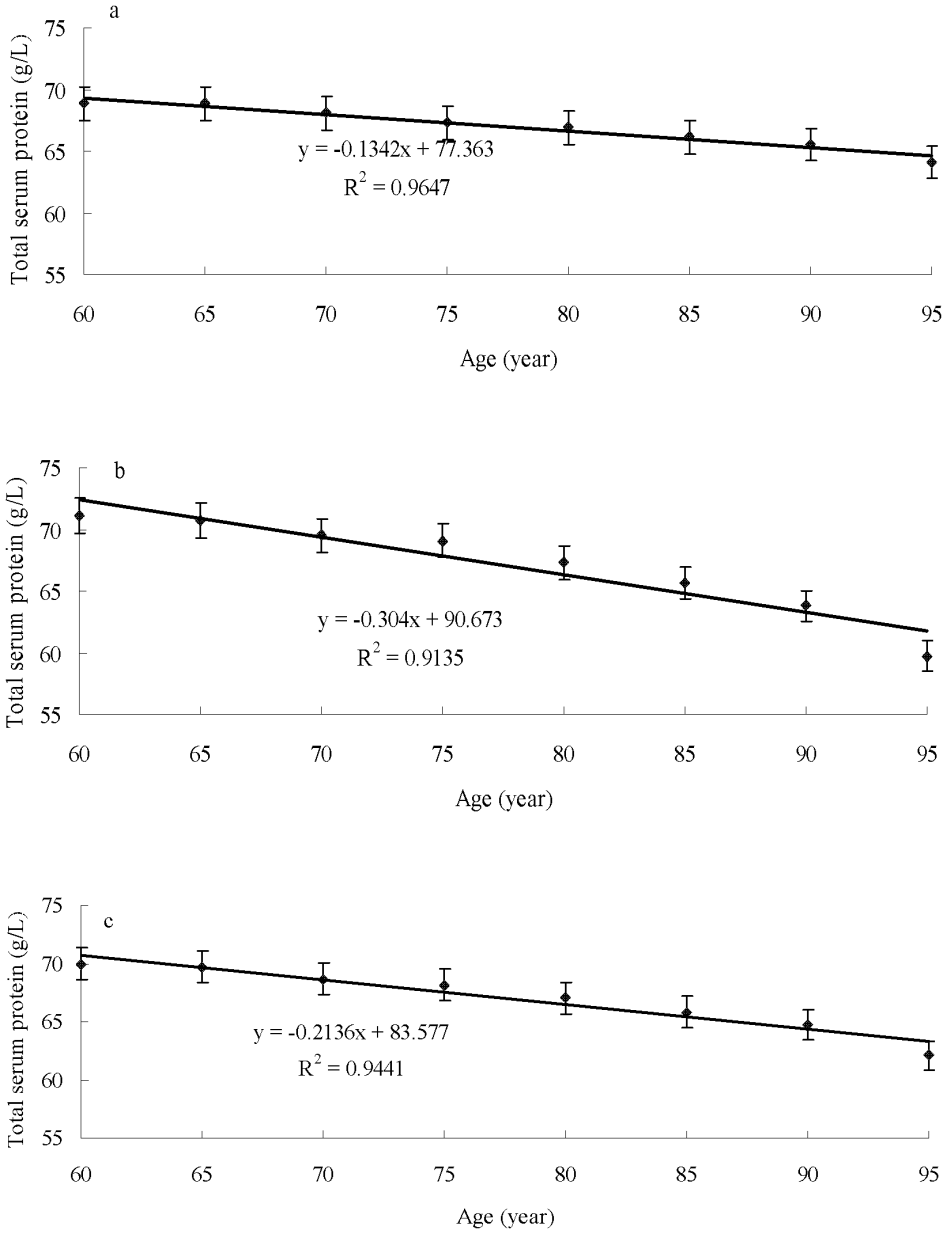
Association of total protein levels with age in male (a), female (b), and overall (c) groups aged ≥60.

The serum total proteins of the male group, female group, and the overall group negatively correlated with age. Regression coefficients were significant in all of the groups (*P*<0.05). The linear regression equation of the total protein with age in the male group was y* = *–0.1342*x* +77.363. Thus, the serum total protein value of males aged ≥60 was reduced by 0.1342 g/L in each additional year (Figure 3a). The linear regression equation of the total protein with age in the female group was *y* = –0.304*x* +90.673. Thus, the total protein value of women aged ≥60 was reduced by 0.304 g/L with each additional year of age (Figure 3b). The linear regression equation of the total protein with age in the overall group was *y* = –0.2136*x* +83.577. Thus, the total protein value of the elderly aged ≥60 was reduced by 0.2136 g/L with each additional year (Figure 3c). Serum total protein levels also decreased faster in females than in males with increasing age (Figure 3).

## Discussion

Serum total protein is of great significance among the elderly. This parameter is closely related to the immune system and maintains intravascular colloid osmotic pressure, hydrogen potential, transportation of various metabolites, and regulation of numerous physiological functions [12]–[15]. Serum total protein levels are mainly determined by liver synthesis; as such, protein levels can be measured to assess nutritional status and help diagnose certain diseases because the loss of proteins can be caused by kidney lesions [16], [17]. For elderly individuals without serious diseases, serum total protein mainly indicates nutritional status and liver function [18], [19].

In the present study, the serum total protein values of 14,453 elderly individual aged ≥60 years exhibited a normal distribution [20]. The reference range of 95% CI was 54.1 g/L to 82.3 g/L in the overall elderly group, 53.7 g/L to 82.0 g/L in males, and 54.8 g/L to 82.8 g/L in females. The reference value for each age group with a five-year group interval is shown in Figure 1. Clear differences were observed between our study and other investigations of serum total protein reference ranges previously reported in China [21], [22]. The differences may be attributed to several factors. First, with an average age of 72.176±7.668 years and a high proportion of individuals aged 80 (19.6%), the age of the subjects in this study is rare for this type of research. Many regional studies of serum total protein have considered age values ranging from 20 years to 60 years. In other studies, subjects aged ≥60 years are often grouped into an entirely new group, and the proportion is usually <10%. Second, regional differences may be important because Lianyungang is a coastal city in eastern China, where Luzhongnan Hills and Huaibei Plain may overlap, and adjacent to the Yellow Sea. Thus, abundant sources of seafood can be obtained in this area than inland, thereby affecting serum total protein levels. Third, a temperature difference is observed between a previous study and Lianyungang, which is located in a warm temperate and subtropical transition zone with an average annual temperature of 14°C. These factors may be accounted for the difference in protein levels observed. Previous reports also demonstrated that total protein levels can be 8% higher when the temperature is >30°C (such as in south China) than that at <0°C (such as in north China) [2]. All of the subjects in this study were in a sitting position during blood collection. Instruments and kits from various manufacturers may also account for differences. In this study, Biuret reaction was performed to determine serum total protein. Biuret reaction is highly accurate and commonly used method in China; however, the bivalent copper ion content and the extent of reaction can vary in terms of different instruments and kits [23]–[26].

This study showed that total protein levels in the elderly females aged between 60 and 79 years were significantly higher than those in males of the same age group (*P*<0.05). No significant difference was observed between the group of males and females aged >80 years; this result is consistent with a previous conclusion stating that the decrease in serum total protein levels in females was significantly faster than in males. The specific reasons for these results remain unclear and a survey with a larger sample should be conducted to elucidate the mechanisms and verify the results presented in this study.

In the present study, serum total protein was negatively correlated with age in the three groups. The regression coefficients were statistically significant (*P*<0.05). In the elderly individuals aged ≥60 years, the value of total protein was reduced by 0.2136 g/L per year. The decreased total protein values in males and females were 0.1342 and 0.304 g/L per year, respectively. One reason for the decrease in serum total protein levels may be aging, in which different stages cause organ dysfunction, particularly the digestive tract, and manifested as loss of appetite, gastrointestinal disorders, slow bowel movements, decreased protein intake, malabsorption, continuously decreasing numbers of liver cells, liver dysfunction, and decreased synthesis of total protein capacity [27], [28]. Second, elderly individuals become weaker and are often susceptible to infections, fever, diarrhea, fractures, and other ailments, which induce the consumption of large amounts of serum protein as age increases [29]–[30]. The present study showed that serum total protein decreased with increasing age in both males and females, but this factor decreased faster in women than in men. Nevertheless, reliable conclusions require a larger sample study for confirmation.

Clinical studies should be conducted to determine whether or not the total protein levels of elderly patients are normal. Serum protein supplements introduced intravenously can reduce mortality and improve the efficacy of therapy in hypoproteinemia. The total plasma protein levels of patients with acute or chronic diseases are correlated negatively with mortality rate [31]–[34]. Previous studies also confirmed that patients with a low serum total protein exhibit poor disease prognosis [35], [36]. Haynes [37] systematically reviewed 79 randomized controlled trials, including 4,755 patients, and demonstrated that the efficacy of treatments can be improved after protein-containing regimens have been administered for patients with heart surgery, ascites, sepsis, burns, and traumatic brain injury. However, non-essential infusion of proteins should be minimized because the rate of protein reabsorption after decomposition is low after serum proteins are administered intravenously, thereby promoting protein decomposition and loss of balance among essential amino acids [38], [39].

In conclusion, the reference range of serum total protein in normal elderly subjects in Lianyungang, China was 54.1 g/L to 82.3 g/L. The total protein levels decreased with increasing age, and the total protein levels decreased faster in women than in men. Considering age and gender differences, we recommend an appropriate serum total protein range for elderly individuals in clinical applications.
